# Changes in Rehmanniae Radix processing and their impact on ovarian hypofunction: potential mechanisms of action

**DOI:** 10.3389/fphar.2024.1426972

**Published:** 2024-07-03

**Authors:** Han-Zhi Zhong, Jing Mo, Yan-Xin Li, Mao-Ya Li, Shao-Bin Wei

**Affiliations:** ^1^ School of Clinical Medicine, Chengdu University of Traditional Chinese Medicine, Chengdu, China; ^2^ Department of Gynecology, Hospital of Chengdu University of Traditional Chinese Medicine, Chengdu, China

**Keywords:** Rehmanniae Radix, medicine processing, pharmacology, traditional Chinese medicine, ovarian hypofunction diseases

## Abstract

**Objective:**

This study evaluates the research developments concerning Rehmanniae Radix in ovarian hypofunction diseases. It explores the processing methods of Rehmanniae Radix, the variations in its compounds before and after processing, the mechanism of Rehmanniae Radix and its active compounds in improving ovarian function, and the advancements in clinical applications of traditional Chinese medicine (TCM) compound that include Rehmanniae Radix.

**Methods:**

Comprehensive literature search was conducted using databases such as China National Knowledge Infrastructure (CNKI), China Science and Technology Journal Database, National Science and Technology Library, the Pharmacopoeia of the People’s Republic of China, Pubmed, and the Web of Science Database. The search utilized the following Medical Subject Headings (MeSH) and keywords: “Rehmanniae Radix,” “Drying Rehmannia Root,” “Rehmannia glutinosa,” “Rehmanniae Radix Praeparata,” “Traditional Chinese Medicine Processing,” “Pharmacological Effects,” “Ovarian Aging,” “Diminished ovarian reserve,” “Premature ovarian insufficiency,” “Premature Ovarian Failure,” “Ovarian hypofunction diseases”.

**Results:**

The ancient Chinese medical books document various processing techniques for Rehmanniae Radix. Contemporary research has identified changes in its compounds processing and the resultant diverse therapeutic effects. When processed into Rehmanniae Radix Praeparata, it is noted for its ability to invigorate the kidney. TCM compound containing Rehmanniae Radix is frequently used to treat ovarian hypofunction diseases, demonstrating significant clinical effectiveness. The key changes in its compounds processing include cyclic dilute ether terpene glycosides, phenylethanol glycosides, sugars, and 5-hydroxymethylfurfural. Its pharmacological action is primarily linked to the improvement of granulosa cell proliferation, antioxidative and anti-aging properties, and modulation of the immune and inflammatory microenvironment. Furthermore, Rehmanniae Radix also offers therapeutic benefits for cardiovascular and cerebrovascular diseases, osteoporosis and cognitive dysfunction caused by low estrogen levels. Thereby Rehmanniae Radix mitigates both the short-term and long-term health risks associated with ovarian hypofunction diseases.

**Conclusion:**

Processed Rehmanniae Radix has shown potential to improve ovarian function, and its compound prescriptions have a definite effect on ovarian dysfunction diseases. Therefore Rehmanniae Radix was garnering interest for both basic and clinical research, with promising application prospects as a future therapeutic agent for ovarian hypofunction diseases. However, further studies on its toxicology and the design of standardized clinical trials are necessary to fully establish its efficacy and safety.

## 1 Introduction

Ovarian hypofunction diseases primarily encompass conditions such as diminished ovarian reserve (DOR), premature ovarian insufficiency (POI), and premature ovarian failure (POF) (Chen et al., 2017). These represent a progressive, gradual decline in ovarian function. DOR primarily highlights the decline in ovarian responsiveness and reproductive function in women of childbearing age, while POI and POF are more focused on the progressive changes associated with age and ovarian aging. Short-term clinical manifestations include menstrual irregularities, reduced fertility and reproductive quality, and perimenopausal symptoms due to low estrogen levels. Menstrual irregularities commonly present as shorter menstrual cycles, prolonged menstrual cycles, reduced menstrual flow, and potentially amenorrhea. A reduction in fertility stems from the progressive decrease in the number of ovarian follicles and a decline in follicle quality, culminating in lower pregnancy rates, higher miscarriage rates, and reduced live birth rates. A decline in reproductive quality is evident in the increased rate of aneuploidy in offspring. The incidence of these conditions is increasingly affecting younger women, significantly impacting female fertility. Reports indicate that the prevalence of DOR ranges from 10% to 32% (Pastore et al., 2018), POI affects about 3.5% (Li M. et al., 2023) of the population, and only approximately 5%–10% (Rebar, 2009) of women diagnosed with POI can conceive spontaneously. However, the impact of ovarian function decline is systemic, posing long-term risks of cardiovascular disease, osteoporosis, and neurological disorders. Estrogen acts as a protective factor for the cardiovascular system, whereas premature ovarian aging markedly elevates the risk of cardiovascular events. Reduced estrogen and elevated FSH levels cause abnormal bone metabolism, leading to osteoporosis and fractures. Furthermore, a decline in ovarian function and changes in HPO axis activity can lead to emotional disturbances, vascular dysfunction, cognitive impairment, and sleep disorders, severely affecting the quality of life for women.

Current treatments primarily involve hormone replacement therapy (HRT) and assisted reproductive technology (ART), with emerging methods such as stem cell therapy still under research. HRT is the primary treatment for alleviating symptoms of low estrogen and providing primary prevention against cardiovascular diseases and osteoporosis. However, HRT cannot restore ovarian function, and its long-term use is associated with risks of thromboembolic diseases, breast tumors, and other adverse effects, prompting concerns regarding the occurrence of adverse reactions (Ewertz et al., 2005; Canonico et al., 2014; Gu et al., 2014). The 2016 European Society of Human Reproduction and Embryology (ESHRE) Guideline advised POI patients with fertility needs to actively pursue ART for conception. Women with a family history of POI or those without current fertility needs may choose to freeze and preserve oocytes or embryos to mitigate age-related fertility decline, thus preserving part of their reproductive potential. Despite advancements in ART, its efficacy is limited in cases of ovarian aging (Leridon, 2004). Meanwhile, as international research on traditional Chinese medicine (TCM) progresses and cross-cultural communication between the East and West deepens, TCM has gained attention due to its natural compounds, multi-target effects, minimal adverse events, and the concept of medicinal and edible use (Heinrich et al., 2021).

Rehmanniae Radix (RR) is either the fresh or dried root of *Rehmannia glutinosa* Libosch., harvested in autumn, cleaned of reeds and fibrous roots, and either used fresh or slow-roasted until it is about 80% dry. Freshly processed RR is termed “Fresh RR (FRR),” and the partially dried form is known as “Raw RR (RRR).” RR has been a staple of TCM for millennia, classed among the “Four Great Medicines,” and is widely used in clinical settings. The processing of RR, which includes methods like steaming and nine steaming and nine drying, transforms it into “RR Praeparata (RRP).” This process alters RR’s medicinal properties from cold to warm and its flavor from bitter to sweet, the function changes from clearing heat to nourishing (Qianfeng, 2005). RR contains various bioactive compounds including iridoid glycosides, polysaccharides, amino acids, and small amounts of β-sitosterol, which undergo changes after processing. RR’s pharmacological actions span multiple systems, offering antioxidant, immune-modulating, anti-inflammatory, and anti-aging benefits, and are principally applied in the treatment of gynecological and diabetic metabolic disorders, cardiovascular diseases, and osteoporosis. This reflects the advantages of TCM in treating complex, multi-system diseases, and functional disorders. It not only fundamentally improves patients’ condition but also offers multi-target synergy and additive effects (Li D. et al., 2023; Zhang, 2023). RR has become a common treatment in clinical settings due to its protective effects on ovary. In recent years, studies on RR’s role in ovarian hypofunction have proliferated, mainly focusing on the mechanism of RR to improve reproductive hormones and increase the number of follicles. At the same time, basic and clinical studies were conducted on the TCM compound containing RR to observe its efficacy, safety and potential mechanism in the treatment of ovarian dysfunction diseases. These studies also seek to explore the long-term health benefits of RR, aiming for synergistic and enhancing effects.

This article reviewed the changes in the compounds of RR before and after processing, its mechanism of action in treating ovarian hypofunction diseases, and the clinical use of related compound prescriptions. It also elaborates on the scientific implications of its transformed medicinal effects to better leverage RR’s therapeutic potential.

## 2 The protective effect of RR on ovary according to TCM theory

RR was first documented in “*Shennong’s Classic Materia Medica”* as “Drying Rehmannia Root” and later appeared as “RRR” in Zhang Zhongjing’s “*Jin Kui Yao Lue”* during the Han Dynasty. The term “RRP” was officially introduced in the Song Dynasty’s “*Bencao Tujing*”. Initially classified as cold, RR’s properties shift to warm after processing, transitioning from an effect of clearing heat to nourishing. This change significantly alters its therapeutic impact on diseases. RRP is the core drug for tonifying the kidney, known for its mild medicinal power and is frequently employed in treating various kidney deficiency diseases in clinical settings. Ancient Chinese medical books extensively describe the clinical signs of ovarian hypofunction diseases, such as menstrual irregularities or amenorrhea and reduced fertility, attributing these conditions to kidney deficiency. According to TCM, the kidney is vital for growth, development, and reproduction, and both menstruation and pregnancy are closely linked to kidney health. The belief that “menstruation originates from the kidney” and “the person who appears amenorrhea before age 49″are supported in “*Fu Qingzhu’s Gynecology,”* aligning with the pathophysiology of these conditions. RRP is acclaimed for its abilities to nourish blood, nourish yin, nourish kidney and invigorate essence. “*Compendium of Materia Medica”* notes that RR has the efficacy in treating “irregular menstruation and long-term infertility.” Furthermore, TCM prescriptions commonly used to treat ovarian hypofunction diseases, such as Guishen Pill, Liuwei Dihuang Pill, Siwu Tang, and Yijing Tang, all incorporate RRP. It can be seen that the clinical treatment of this type of disease is inseparable from the use of RRP.

## 3 Changes in the compounds of RR before and after processing

### 3.1 Methods of processing

Archaeological research has revealed that processed RR found in the Haihunhou tomb from the Western Han Dynasty is the earliest Chinese medicine excipient concoction product found in ancient China so far, which involved excipients of starch and sucrose, suggesting that these samples were processed by rice steaming (Peng et al., 2019; Zhu et al., 2021). The history of RR processing involves a variety of methods. Traditional methods include steaming and boiling without excipients, as well as the excipients of ingredients such as alcohol, Amomi Fructus and ginger (Xie et al., 2022). In contemporary practice, processed RR varieties with region-specific characteristics have developed, such as Wenzhi RR with Jiangxi Jianchang Band and Salt RR from Yunnan.

Historically, RR has been known by various names depending on the processing method employed. However, the 2020 edition of *The Pharmacopoeia of the People’s Republic of China* recognizes only two designations: RR and RRP. RR encompasses Fresh RR and RRR, with RRP defined as the processed product of RRR, the specific two processing methods are steaming and wine stewing. In addition, “nine steamed and nine sun-dried” is also a traditional processing method of RR.

Steamed refers to the method of heating the raw productswithout excipients and separating water to a certain extent. The description of the 2020 edition of *The Pharmacopoeia of the People’s Republic of China* is more precise, which points that “steamed with water involves heating the raw medicinal botanical drug by placing it over boiling water until it achieves a black and moist state. It is then removed and dried to about eighty percent dryness before being cut into thick slices or chunks and fully dried to produce RRP” (National Pharmacopoeia Committee, 2020).

The method of stewing with wine, widely used today, was first documented in *Shennong Bencao Jing Jizhu* during the Liang Dynasty. *Leigong Paozhi Lun* during the Northern and Southern Dynasties mentioned for the first time that wine was used as an auxiliary material to mix and steam. This method entails mixing RR with wine prior to steaming (Wang et al., 2019). The pharmacopeia (National Pharmacopoeia Committee, 2020) details that for every 100 kg of RRR, 30–50 kg of yellow rice wine is used. The operational steps include stewing the RR with wine until it fully absorbs the wine, followed by air drying until the sticky fluid on its surface slightly dries. It is then processed further into thick slices or chunks to finally obtain RRP.

The “nine steamed and nine sun-dried” method, with a long historical lineage, first appeared in the Tang Dynasty’s *Qianjin Yi Fang* and was subsequently referenced in later texts such as the Song Dynasty’s *Bencao Tujing*, the Yuan Dynasty’s *Tangye Bencao*, the Ming Dynasty’s *Bencao Gangmu.* While the exact number of steaming and sun-drying cycles described in these books may vary, the overall goal is to control the quality of RRP, indicating that the number of cycles is not rigidly fixed (Yan et al., 2020; Wu et al., 2022). It is theorized that the number “nine” in “nine steamed and nine sun-dried” reflects a traditional practice where some Chinese medicines are often processed this way multiple times, thus giving rise to the collective term (Peng et al., 2018).

### 3.2 Active compounds before processing

**FIGURE 1:**
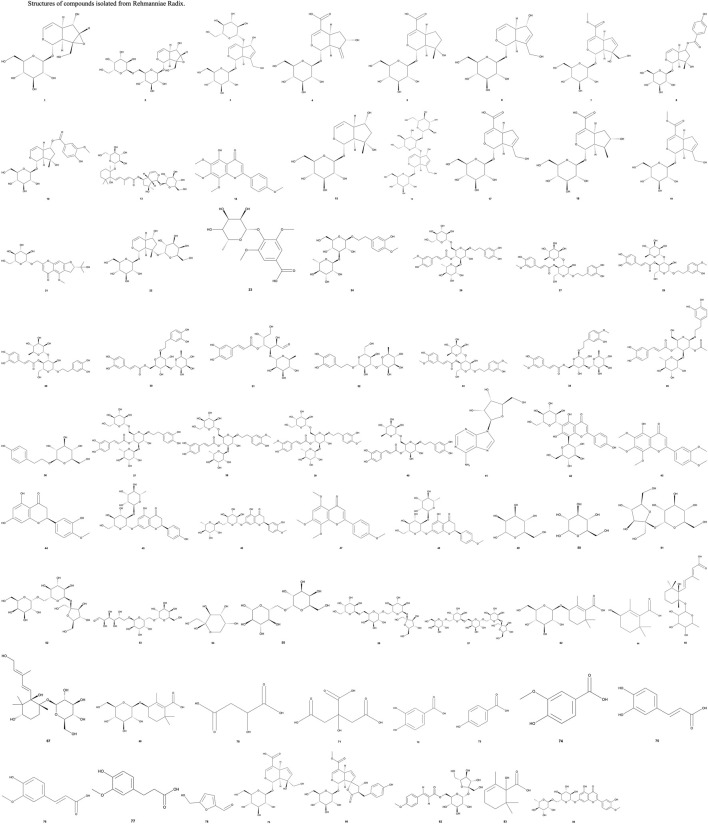
Structures of compounds isolated from Rehmanniae Radix. P.S: The structures in this article were referenced from the PubChem database. Structures not displayed were not found in the database. The bold Arabic numbers below the structures in Figure 1 correspond to the compounds numbered in Table 1.

*The Pharmacopoeia of the People’s Republic of China* identifies rehmannioside D and catalpol, both iridoid glycosides, as the key indicators for assessing the quality of RRR. Currently, 75 compounds have been extracted from RRR, which are categorized into classes such as iridoid glycosides, phenylethanoid glycosides, flavonoids, ionones, phenolic acids, and carbohydrates. The structures of these compounds are illustrated in Figure 1. Among these, iridoid glycosides, phenylethanoid glycosides, carbohydrates, and nucleosides are frequently used as reference compounds for selecting quality markers for RR (Zhu J. et al., 2022). These compounds are consolidated into Table 1 (Li et al., 2020; Gu et al., 2021; Chen SQ. et al., 2023; Lu et al., 2023; Xue et al., 2023; Zhu et al., 2023).

**TABLE 1:** Compounds in raw Rehmanniae Radix and Rehmanniae Radix praeparata.

No.	Category	Compound	Molecular formulas	Extraction method	Raw	Steamed	Wine stewed	Repeatedly steamed and sundried	References
1	iridoid glycosides	Catalpol	C_15_H_22_O_10_	methanol, by ultrasonication	+	NR	NR	+	Gu et al. (2021), Chen et al. (2023a)
2	iridoid glycosides	rehmannioside A	C_21_H_32_O_15_	methanol, by ultrasonication	+	+	+	-	Li et al. (2020), Gu et al. (2021)
3	iridoid glycosides	Melittoside	C_21_H_32_O_15_	methanol, by ultrasonication	+	+	+	-	Lu et al. (2023)
4	iridoid glycosides	Gardoside	C_16_H_22_O_10_	methanol, by ultrasonication	+	+	+	+	Lu et al. (2023)
5	iridoid glycosides	mussaenosidic acid	C_16_H_24_O_10_	methanol, by ultrasonication	+	+	+	+	Lu et al. (2023)
6	iridoid glycosides	Rhinanthin	C_15_H_22_O_9_	methanol, by ultrasonication	+	+	+	+	Lu et al. (2023)
7	iridoid glycosides	Gardenoside	C_17_H_24_O_11_	methanol, by ultrasonication	+	+	+	+	Lu et al. (2023)
8	iridoid glycosides	6-O-p-hydroxybenzoyl ajugol	C_22_H_28_O_11_	methanol, by ultrasonication	+	-	-	-	Lu et al. (2023)
9	iridoid glycosides	6-O-E-caffeoyl ajugol	C_24_H_30_O_12_	methanol, by ultrasonication	+	+	+	-	Lu et al. (2023)
10	iridoid glycosides	6-O-Vanilloylajugol	C_23_H_30_O_12_	methanol, by ultrasonication	+	+	+	-	Lu et al. (2023)
11	iridoid glycosides	6-O-p-Vanillyl Ajugaol	C_24_H_30_O_11_	methanol, by ultrasonication	+	+	+	+	Lu et al. (2023)
12	iridoid glycosides	6-O-E-feruloyl Ajugaol	C_25_H_32_O_12_	methanol, by ultrasonication	+	+	+	+	Lu et al. (2023)
13	iridoid glycosides	rehmaglutoside I	C_36_H_56_O_17_	methanol, by ultrasonication	+	-	-	-	Lu et al. (2023)
14	iridoid glycosides	gardenin B	C_19_H_18_O_7_	methanol, by ultrasonication	-	-	-	-	Lu et al. (2023)
15	iridoid glycosides	Leonuride	C_15_H_24_O_9_	methanol, by ultrasonication	+	+	+	+	Gu et al. (2021), Chen et al. (2023a), Lu et al. (2023)
16	iridoid glycosides	rehmannioside D	C_27_H_42_O_20_	methanol, by ultrasonication	+	+	+	+	Gu et al. (2021), Chen et al. (2023a), Lu et al. (2023)
17	iridoid glycosides	geniposidic acid	C_16_H_22_O_10_	methanol, by ultrasonication	+	+	+	+	Lu et al. (2023), Zhu et al. (2023)
18	iridoid glycosides	8-epiloganic acid	C_16_H_24_O_10_	methanol, by ultrasonication	+	+	+	+	Lu et al. (2023), Zhu et al. (2023)
19	iridoid glycosides	Geniposide	C_17_H_24_O_10_	methanol, by ultrasonication	+	NR	+	+	Zhu et al. (2023)
20	iridoid glycosides	Acetylcatalpol	C_17_H_24_O_11_	methanol, by ultrasonication	+	NR	+	+	Zhu et al. (2023)
21	iridoid glycosides	prim-o-glucosylcimifugin	C_22_H_28_O_11_	methanol, by ultrasonication	+	NR	+	+	Zhu et al. (2023)
22	iridoid glycosides	rehmannioside C	C_19_H_32_O_8_	methanol, by ultrasonication	+	NR	+	+	Zhu et al. (2023)
23	phenylethanoid glycosides	syringic acid-4-O-α-L rhamnoside	C_15_H_20_O_9_	methanol, by ultrasonication	+	+	+	-	Lu et al. (2023)
24	phenylethanoid glycosides	darendoside B	C_21_H_32_O_12_	methanol, by ultrasonication	+	+	+	+	Lu et al. (2023)
25	phenylethanoid glycosides	2-phenylethyl-O-β-D xylopyranosyl-(1→6)-β-D-glucopyranoside	C_19_H_28_O_10_	methanol, by ultrasonication	+	+	+	+	Lu et al. (2023)
26	phenylethanoid glycosides	jionoside A_1_/A_2_	C_36_H_48_O_20_	methanol, by ultrasonication	+	+	+	+	Lu et al. (2023)
27	phenylethanoid glycosides	leucosceptoside A	C_30_H_38_O_15_	methanol, by ultrasonication	+	+	+	+	Lu et al. (2023)
28	phenylethanoid glycosides	jionoside D	C_30_H_38_O_15_	methanol, by ultrasonication	+	+	+	+	Lu et al. (2023)
29	phenylethanoid glycosides	Verbascoside	C_29_H_36_O_15_	methanol, by ultrasonication	+	+	+	+	Gu et al. (2021), Chen et al. (2023a), Lu et al. (2023)
30	phenylethanoid glycosides	Isoverbascoside	C_29_H_36_O_15_	methanol, by ultrasonication	+	+	+	+	Chen et al. (2023a), Lu et al. (2023), Zhu et al. (2023)
31	phenylethanoid glycosides	cistanoside F	C_21_H_28_O_13_	methanol, by ultrasonication	+	+	+	+	Lu et al. (2023), Zhu et al. (2023)
32	phenylethanoid glycosides	Verbasoside	C_20_H_30_O_12_	methanol, by ultrasonication	+	+	+	+	Lu et al. (2023), Zhu et al. (2023)
33	phenylethanoid glycosides	Martynoside	C_31_H_40_O_15_	methanol, by ultrasonication	+	+	+	+	Lu et al. (2023), Zhu et al. (2023)
34	phenylethanoid glycosides	Isomartynoside	C_31_H_40_O_15_	methanol, by ultrasonication	+	+	+	+	Lu et al. (2023), Zhu et al. (2023)
35	phenylethanoid glycosides	2′-acetylacteoside	C_31_H_38_O_16_	methanol, by ultrasonication	+	NR	+	+	Zhu et al. (2023)
36	phenylethanoid glycosides	Rhodioloside	C_14_H_20_O_7_	methanol, by ultrasonication	+	NR	+	+	Zhu et al. (2023)
37	phenylethanoid glycosides	Echinacoside	C_35_H_46_O_20_	methanol, by ultrasonication	+	NR	+	+	Zhu et al. (2023)
38	phenylethanoid glycosides	cistanoside a	C_36_H_48_O_20_	methanol, by ultrasonication	+	NR	+	+	Zhu et al. (2023)
39	phenylethanoid glycosides	jionoside b1	C_37_H_50_O_20_	methanol, by ultrasonication	+	NR	+	+	Zhu et al. (2023)
40	phenylethanoid glycosides	forsythiaside A	C_29_H_36_O_15_	methanol, by ultrasonication	+	NR	+	+	Zhu et al. (2023)
41	nucleosides	Adenosine	C_10_H_13_N_5_O_4_	methanol, by ultrasonication	+	NR	+	+	Zhu et al. (2023)
42	flavonoids	vicenin Ⅱ	C_27_H_30_O_15_	methanol, by ultrasonication	-	-	-	-	Lu et al. (2023)
43	flavonoids	Demethylnobiletin	C_20_H_20_O_8_	methanol, by ultrasonication	-	-	-	-	Lu et al. (2023)
44	flavonoids	Hesperetin	C_16_H_14_O_6_	methanol, by ultrasonication	-	-	-	-	Lu et al. (2023)
45	flavonoids	Naringin	C_27_H_32_O_14_	methanol, by ultrasonication	-	-	-	-	Lu et al. (2023)
46	flavonoids	hesperidin/neohesperidin	C_28_H_34_O_15_	methanol, by ultrasonication	-	-	-	-	Lu et al. (2023)
47	flavonoids	6-demethoxytangeretin	C_19_H_18_O_6_	methanol, by ultrasonication	-	-	-	-	Lu et al. (2023)
48	flavonoids	Poncirin	C_28_H_34_O_14_	methanol, by ultrasonication	-	-	-	-	Lu et al. (2023)
49	carbohydrates	Mannose	C_6_H_12_O_6_	Petroleum ether and ethyl acetate were extracted twice each	-	NR	NR	+	Xue et al. (2023)
50	carbohydrates	Glucose	C_6_H_12_O_6_	Petroleum ether and ethyl acetate were extracted twice each	+	NR	NR	+	Xue et al. (2023)
51	carbohydrates	Sucrose	C_12_H_22_O_11_	Petroleum ether and ethyl acetate were extracted twice each	+	NR	NR	-	Xue et al. (2023)
52	carbohydrates	Raffinose	C_18_H_32_O_16_	Petroleum ether and ethyl acetate were extracted twice each	+	NR	NR	-	Xue et al. (2023)
53	carbohydrates	Manninotriose	C_18_H_32_O_16_	Petroleum ether and ethyl acetate were extracted twice each	+	NR	NR	+	Xue et al. (2023)
54	carbohydrates	Fructose	C_6_H_12_O_6_	Petroleum ether and ethyl acetate were extracted twice each	+	NR	+	+	Xue et al. (2023), Zhu et al. (2023)
55	carbohydrates	Melibiose	C_12_H_22_O_11_	Petroleum ether and ethyl acetate were extracted twice each	-	NR	+	+	Xue et al. (2023), Zhu et al. (2023)
56	carbohydrates	Stachyose	C_24_H_42_O_21_	Petroleum ether and ethyl acetate were extracted twice each	+	NR	-	-	Xue et al. (2023), Zhu et al. (2023)
57	carbohydrates	Verbascose	C_30_H_52_O_26_	methanol, by ultrasonication	+	NR	+	+	Zhu et al. (2023)
58	lonones	rehmannia neoterpene B	C_15_H_24_O_6_	methanol, by ultrasonication	+	+	+	+	Lu et al. (2023)
59	lonones	rehmannia neoterpene E	C_21_H_34_O_10_	methanol, by ultrasonication	+	+	+	+	Lu et al. (2023)
60	lonones	hydroxyl wild acid	C_15_H_24_O_5_	methanol, by ultrasonication	+	+	+	+	Lu et al. (2023)
61	lonones	rehmannia neoterpene F	C_21_H_34_O_10_	methanol, by ultrasonication	+	+	+	+	Lu et al. (2023)
62	lonones	Rehmapicroside	C_16_H_26_O_8_	methanol, by ultrasonication	+	+	+	+	Lu et al. (2023)
63	lonones	oxyrehmanionoside B	C_19_H_34_O_9_	methanol, by ultrasonication	+	+	+	+	Lu et al. (2023)
64	lonones	Rehmapicrogenin	C_10_H_16_O_3_	methanol, by ultrasonication	+	+	+	+	Lu et al. (2023)
65	lonones	aeginetic acid 5-O-β-D quinovoside	C_21_H_34_O_8_	methanol, by ultrasonication	+	+	+	+	Lu et al. (2023)
66	lonones	rehmannia neoterpene C	C_28_H_40_O_10_	methanol, by ultrasonication	+	+	+	+	Lu et al. (2023)
67	lonones	neo-rehmannioside	C_21_H_36_O_9_	methanol, by ultrasonication	+	+	+	+	Lu et al. (2023)
68	lonones	kojic acid	C_15_H_24_O_4_	methanol, by ultrasonication	+	+	+	+	Lu et al. (2023), Zhu et al. (2023)
69	lonones	rehmapicroside or its isomers	C_16_H_26_O_8_	methanol, by ultrasonication	+	NR	+	-	Zhu et al. (2023)
70	organic acids	malic acid	C_4_H_6_O_5_	methanol, by ultrasonication	+	+	+	+	Lu et al. (2023)
71	organic acids	citric acid	C_6_H_8_O_7_	methanol, by ultrasonication	+	+	+	+	Lu et al. (2023)
72	phenolic acids	protocatechuic acid	C_7_H_6_O_4_	methanol, by ultrasonication	+	+	+	+	Lu et al. (2023)
73	phenolic acids	p-hydroxybenzoic acid	C_7_H_6_O_3_	methanol, by ultrasonication	+	+	+	+	Lu et al. (2023)
74	phenolic acids	vanillic acid	C_8_H_8_O_4_	methanol, by ultrasonication	+	+	+	+	Lu et al. (2023)
75	phenolic acids	caffeic acid	C_9_H_8_O_4_	methanol, by ultrasonication	+	+	+	+	Lu et al. (2023)
76	phenolic acids	ferulic acid	C_10_H_10_O_4_	methanol, by ultrasonication	+	+	+	+	Lu et al. (2023)
77	phenolic acids	dihydroferulic acid	C_10_H_12_O_4_	methanol, by ultrasonication	+	NR	+	+	Zhu et al. (2023)
78	others	5-hydroxymethyl-2 -furfura	C_6_H_6_O_3_	methanol, by ultrasonication	-	+	+	+	Li et al. (2020), Zhu et al. (2023)
79	others	Monotropeine	C_16_H_22_O_11_	methanol, by ultrasonication	+	+	+	+	Lu et al. (2023)
80	NR	Dunnisinoside	C_26_H_30_O_13_	methanol, by ultrasonication	+	+	+	+	Lu et al. (2023)
81	NR	rhamnopyranosyl vanilloyl	C_14_H_18_O_8_	methanol, by ultrasonication	+	+	+	+	Lu et al. (2023)
82	NR	sibirioside B	C_22_H_30_O_13_	methanol, by ultrasonication	+	+	+	+	Lu et al. (2023)
83	NR	1-hydroxy-2,6,6-trimethylcyclohex-2-enecarboxylic acid	C_10_H_16_O_3_	methanol, by ultrasonication	+	+	+	+	Lu et al. (2023)
84	NR	Diosmin	C_28_H_32_O_15_	methanol, by ultrasonication	-	-	-	-	Lu et al. (2023)
85	NR	gionoside C	C_29_H_36_O_13_	methanol, by ultrasonication	+	+	+	+	Lu et al. (2023)
86	NR	jiocarotenoside A_1_/A_2_	C_21_H_34_O_9_	methanol, by ultrasonication	+	+	+	+	Lu et al. (2023)
87	NR	nigroside Ⅰ/Ⅱ	C_30_H_38_O_14_	methanol, by ultrasonication	+	+	+	+	Lu et al. (2023)

P.S: +: detected in samples; -: can’t be found in samples; NR: no researches were carried out. The references cited in the table are [22-26、32] and are listed below.

### 3.3 Active compounds after processing

RRP undergoes numerous processing methods, each affecting its compounds and concentration differently. Modern studies focus on compounds like iridoid glycosides, phenylethanoid glycosides, carbohydrates, and 5-hydroxymethyl furfural (5-HMF). Table 1 details the extraction methods and the compounds derived from both RRR and RRP, processed respectively by steaming with water, stewing with wine, and repeated steamed and sun-dried.

#### 3.3.1 Glycoside analysis

In RRP, the glycoside compounds most researched are iridoid glycosides, phenylethanoid glycosides, and nucleosides.

Experimental studies reveal iridoid glycosides and phenylethanoid glycosides as significant quality markers, reflecting the degree of processing in RRP (Liu et al., 2023). The experimental data showed that the content of rehmannioside D after steaming and wine stewing was lower than that before processing, and the content of rehmannioside D in wine stewing was less than that of steamed products with the same degree of processing (Jiang Y. et al., 2023). Using UPLC-PDA and HPLC methods for testing (Zhang LX. et al., 2023; Yang et al., 2024), the data shows that in the process of wine steaming, a significant decrease was observed in iridoid glycosides such as catalpol, rehmannioside D, rhinanthin, leonuride, geniposidic acid, and melittin during wine stewing. The most substantial decline was in catalpol, dropping from 44.00 mg/g in FRR to 2.192 mg/g in the wine stewed RR. Similarly, verbascoside from phenylethanoid glycosides showed a notable reduction during wine stewing. In nucleosides, adenosine and uridine levels increased, whereas guanosine decreased. Further HPLC analysis indicated that with successive steaming from one to nine times, the concentrations of catalpol, rehmannioside D, leonuride, and verbascoside diminished (Ma et al., 2023). However, during the “nine steamed and nine sun-dried” process, from “one steamed and one sun-dried” to “seven steamed and seven sun-dried,” the concentration of catalpol, rehmannioside D, leonuride, verbascoside and isoverbascoside increased with the number of cycles, yet showed no significant change from “seven steamed and seven sun-dried” to “nine steamed and nine sun-dried” (Chen SQ. et al., 2023). Experimental findings (Zhu et al., 2023) suggest that compared to RRR, the total iridoid glycoside concentration decreased in three processed forms: RR stewed with wine, nine steamed and nine sun-dried RR (without Citri Reticulatae Pericarpium and Amomi Fructus), and nine steamed and nine sun-dried RR (with Citri Reticulatae Pericarpium and Amomi Fructus). Conversely, the total phenylethanoid glycoside concentration increased in the nine steamed and nine sun-dried RRP (with Citri Reticulatae Pericarpium and Amomi Fructus) while decreasing in RR steamed with wine, RR stewed with wine, and nine steamed and nine sundried RR (without Citri Reticulatae Pericarpium and Amomi Fructus). Other studies (Chen et al., 2021) have noted a general decrease in glycoside concentration after processing, particularly higher levels of rehmannioside D, heterophylloside, and leonuride in RR processed with both Citri Reticulatae Pericarpium and Amomi Fructus compared to processing with just one of these excipients.

#### 3.3.2 Carbohydrates

Experiments demonstrate that carbohydrate components exhibit different trends over the course of processing. These variations not only distinguish between different processed RR, but also, to some extent, indicate the processing endpoint for RRP, providing a partial reference for its quality evaluation. (Zhang et al., 2020).

The ATR-FTIR method was utilized to conduct a comprehensive analysis of water extracts from FRR, RRR, and RRP, quantifying compounds such as mannose, manninotriose, stachyose, fructose, glucose, galactose, melibiose, and verbascoside. This confirms that carbohydrates are the primary differential compounds between FRR and its processed forms (Tian et al., 2022). Quantitative analysis at various processing stages shows that while sucrose, raffinose, stachyose, and verbascoside decrease, fructose, glucose, melibiose, and manninotriose increase significantly. Carbohydrate profiles in RRR and RRP thus exhibit distinct characteristics, forming a basis for quality assessment of both (Zhang et al., 2016). During wine stewing and steaming, polysaccharide concentration initially rises then falls. The polysaccharide content in wine-stewed products is higher than that in steamed products at the same processing stage (Jiang Y. et al., 2023). During the “one steamed and one sun-dried” to “seven steamed and seven sun-dried” processing, polysaccharide concentration in RRP increases with each cycle. However, from “seven steamed and seven sun-dried” to “nine steamed and nine sun-dried,” the polysaccharide concentration declines. The highest polysaccharide mass fraction, 30.10%, is found in the “seven steamed and seven sun-dried” RRP (Chen SQ. et al., 2023). During nine steaming cycles, the polysaccharide concentration in RRR is lowest, peaking in the fifth cycle before sharply declining, the variation in polysaccharide content in RRP after six steaming cycles is minimal (Jia et al., 2023). By measuring the content of D-fructose, glucose, sucrose, melibiose, stachyose, manninotriose, raffinose, and verbascoside, it was found that after processing trends show a decrease in oligosaccharide concentration and an increase in monosaccharide concentration. In RR processed with Amomi Fructus and Citri Reticulatae Pericarpium, the concentration of D-fructose, glucose, and manninotriose increased by 29.24%, 57.14%, and 44.65% respectively, compared to samples processed without these excipients (Chen et al., 2021).

#### 3.3.3 Other compounds

**FIGURE 2:**
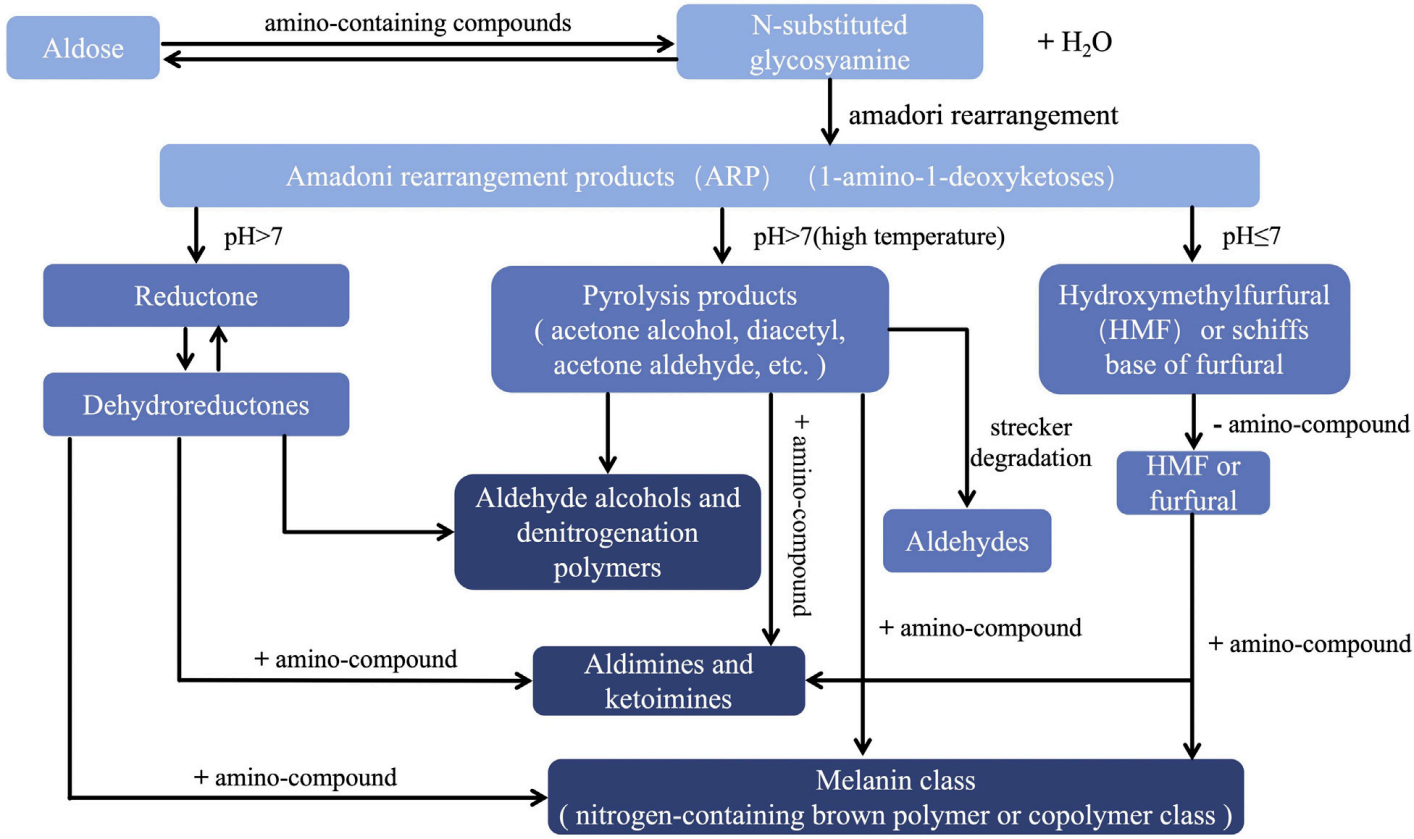
Schematic diagram of Hodge Maillard reaction process. PS: The color from light to dark corresponds to the initial stage, intermediate stage and final stage of the Maillard reaction, respectively. [40] Gong RZ, Huo XH, Zhang L, Liu C, Li SS, Sun YS. (2019). Advances in effects and regulation of Maillard reaction on quality of Chinese materia medica. Chinese Traditional and Herbal Drugs (01),243-251.

5-HMF, a marker of the Maillard reaction (Wang et al., 2020), is a new substance formed during RR processing. The mechanism and products of the Maillard reaction are highly complex. When Hodge named this compound reaction in 1953, he summarized the process of the reaction (Shown in the Figure 2 (Gong et al., 2019; Zhou et al., 2014). It is absent in RRR but its concentration significantly increases after processing (Chen et al., 2021; Zhu et al., 2023), with its presence correlating strongly with the darkening color of RRP (Ma et al., 2023). In addition, it has also been found in the processing studies of other traditional Chinese medicines that the presence of 5-HMF can be detected after processing, but not before processing, indicating that 5-HMF can be used as an indicator to evaluate the degree of processing (Yang et al., 2023).

Trace element analysis from “one steamed and one sun-dried” to “nine steamed and nine sun-dried” shows an initial increase and then a decrease in beneficial elements like zinc, copper, and iron, while harmful elements such as cadmium, arsenic, and lead gradually decline with more steaming and sun-drying cycles, potentially reducing the accumulation of toxic substances in human soft tissues (Chen SQ. et al., 2023).

Amino acid analysis revealed that both total and free amino acid mass fractions decrease in processed RR, with basic amino acids, especially arginine and lysine, showing the most significant declines (Gao et al., 2010).

## 4 Application of RR and its active compounds in ovarian hypofunction diseases

**FIGURE 3:**
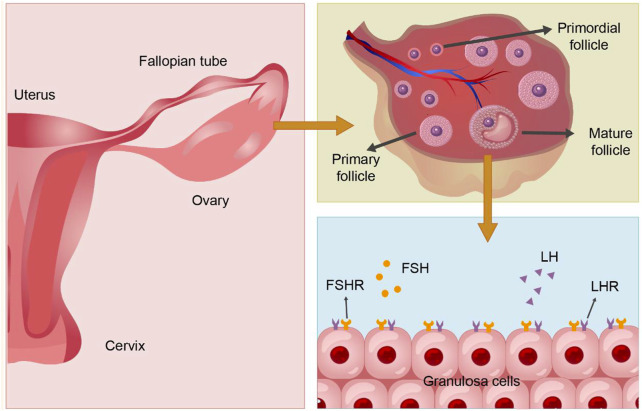
The structure and physiology of the ovary and granulosa cells. PS: Follicle stimulating hormone (FSH), Follicle stimulating hormone receptor (FSHR); Luteinizing hormone (LH), Luteinizing hormone receptor (LHR).

RR exhibits a range of pharmacological effects. *In vivo* and *in vitro* experiments have confirmed its mechanism in enhancing granulosa cell proliferation, antioxidant and anti-aging effects, and modulation of the immune-inflammatory microenvironment. Thus, it plays a therapeutic role in ovarian hypofunction diseases, with reproductive endocrine protection function. The mechanism of Ovarian Hypofunction Diseases and the function of RR are shown in Figure 3.

### 4.1 Improves granulosa cell proliferation

**FIGURE 4:**
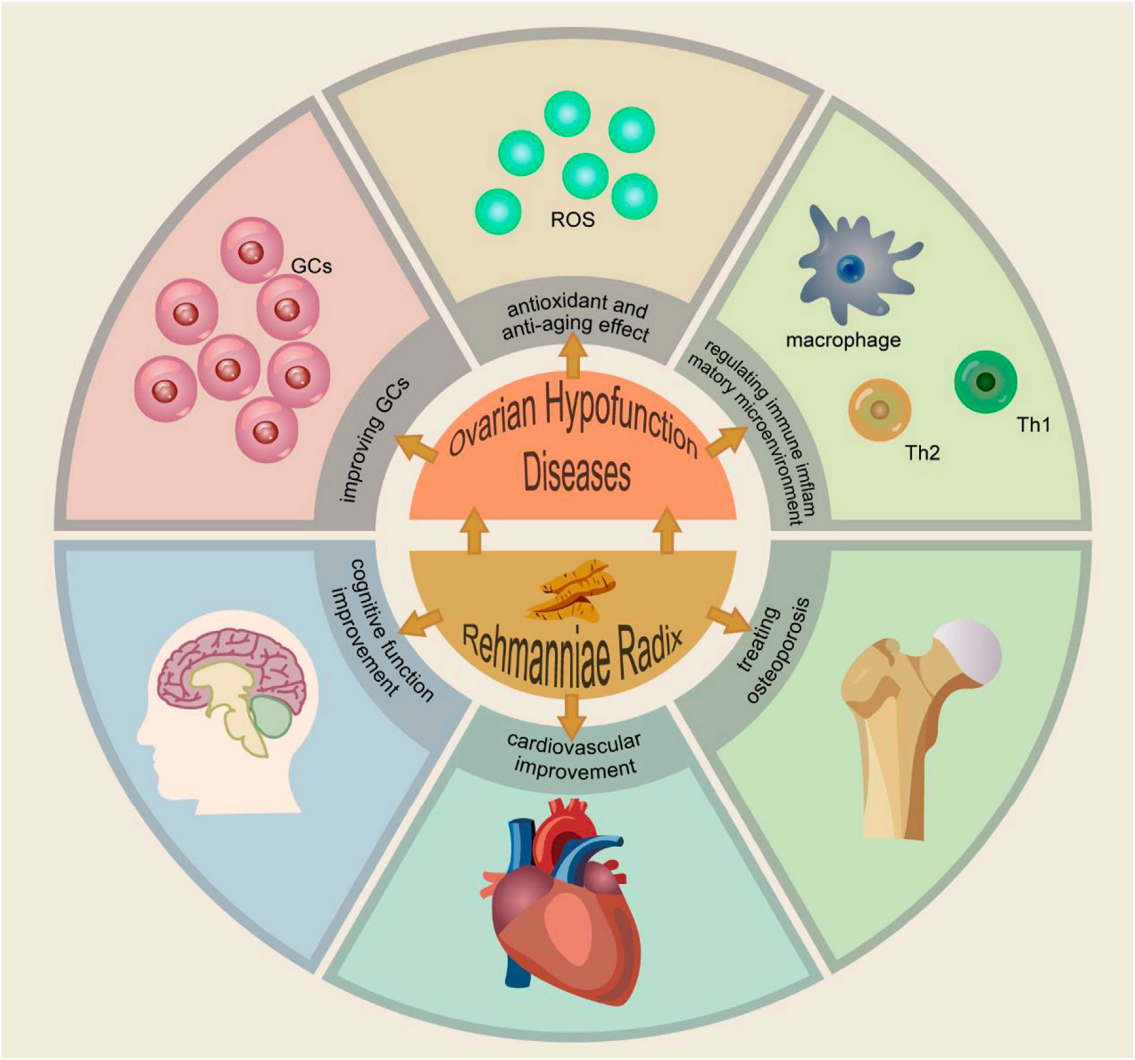
The Mechanism of Ovarian Hypofunction Diseases and the Function of Rehmanniae Radix. PS: Granulosa cells (GCs). Reactive oxygen species (ROS).

**FIGURE 5:**
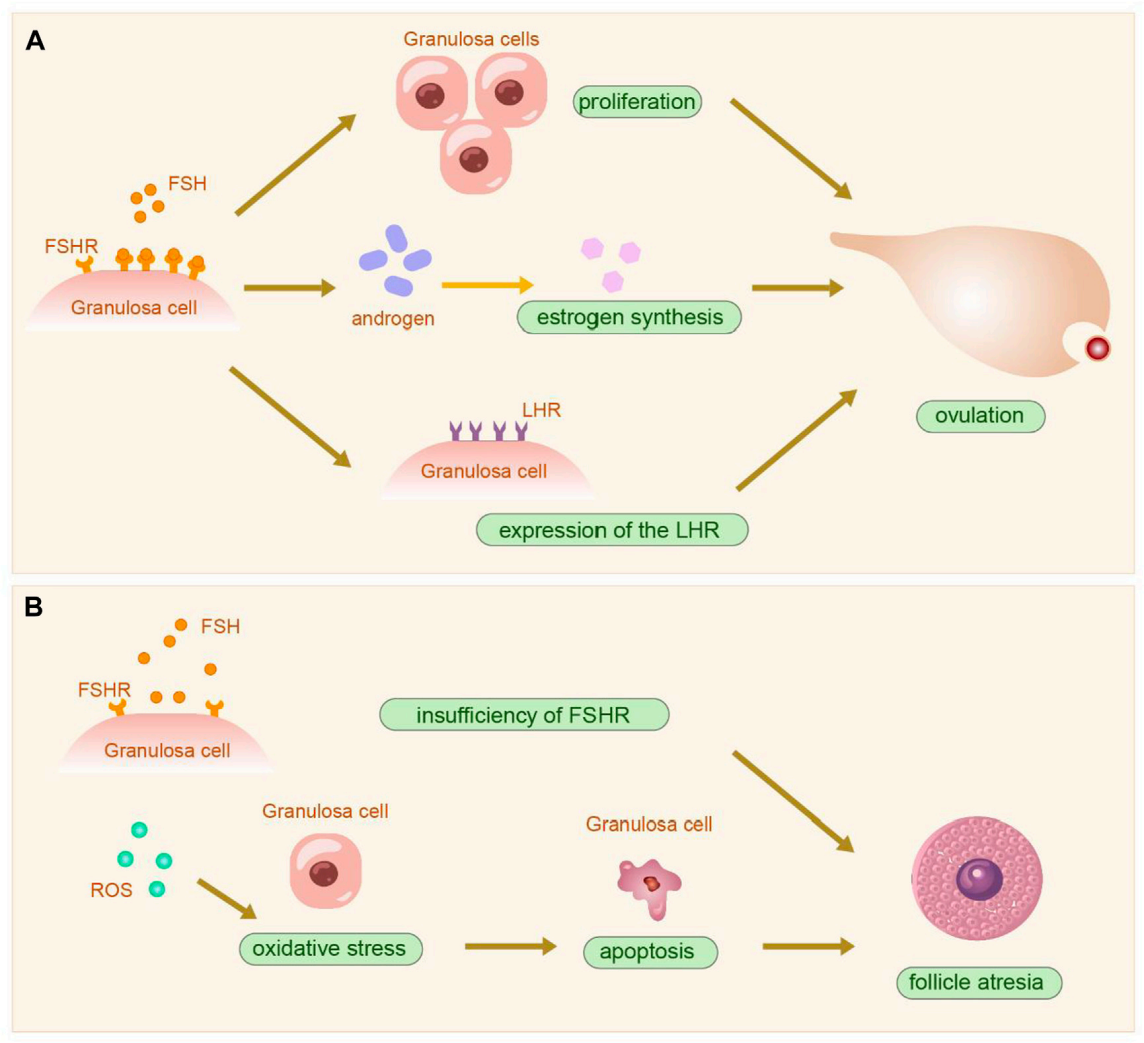
The physiopathological diagram of granulosa cells. **(A)**. The physiological process following the action of FSH on ovarian granulosa cells. **(B)**. The onset of follicle atresia after insufficiency of FSHR and oxidative stress. PS: Follicle stimulating hormone (FSH); Follicle stimulating hormone receptor (FSHR); Luteinizing hormone receptor (LHR); Reactive oxygen species (ROS).

The follicle, composed of oocytes, granulosa cells (GCs), and follicular membrane cells, is fundamental to ovarian function. The proliferation and differentiation of GCs signify follicle development. GCs are crucial for synthesizing, expressing, and metabolizing various hormones, serving as the primary source of ovarian estradiol, inhibin, and activin, and playing roles in oocyte reserve, maturation, and pregnancy maintenance (Kranc et al., 2017). The structure and physiology of the ovary and GCs are shown in Figure 4. GCs are central to studies on follicular growth and atresia mechanisms. Ovarian hypofunction diseases relate to GC apoptosis, follicle stimulating hormone (FSH) receptor signaling defects, autoimmunity, and other factors (Nelson, 2009). Prior studies suggest that abnormal proliferation and apoptosis of ovarian GCs may cause excessive follicular atresia (Matsuda et al., 2012). Additionally, hormones from the pituitary gland and ovaries regulate ovarian function and follicle development and atresia. FSH initiates key events in ovarian follicles: GC proliferation, estrogen synthesis, and expression of the luteinizing hormone receptor (LHR) in GCs (Liu et al., 2024), crucial for GC differentiation in antral and preovulatory follicles (Hsueh et al., 2000). The follicle stimulating hormone receptor (FSHR), a specific G protein-coupled receptor on ovarian GCs (Hunzicker-Dunn and Maizels, 2006), promotes GCs proliferation by binding to FSH, facilitates androgen conversion to estrogen, leads to follicle maturation, produces a peak in LH levels, and induces ovulation (Casarini and Crépieux, 2019) (Shown in the Figure 5). Haploinsufficiency of FSHR accelerates oocyte loss in mice, marking a significant factor in ovarian aging and estrogen deficiency (Danilovich and Sairam, 2002).

RRP is commonly used to tonify the kidney to promote follicle development and maturation, but the effects on ovaries vary with different processing methods and degrees. Researchers (Que et al., 2021) have observed varying impacts of the nine steamed and nine sun-dried RRP group, the one steamed and one sun-dried RRP group, and the RRR group on mouse ovaries. The nine steamed and nine sun-dried RRP group significantly increased preantral and sinusoid follicles and elevated FSHR levels in the ovaries, unlike the one steamed and one sun-dried RRP group and the RRR group, which did not promote follicle development. FSHR may be a target of the nine steamed and nine sun-dried RRP. Furthermore, 5-HMF, a compound formed by the Maillard reaction of sugars and amino acids during processing, was identified as potentially influencing the medicinal properties of RRP. The concentration of 5-HMF correlates closely with steaming and heating time (Yang, 2010). Enhanced expression of FSHR improves GCs function, while silencing FSHR expression leads to GCs apoptosis and follicular atresia (Du et al., 2016). It has been suggested from experimental studies that processed RR might increase 5-HMF concentration and upregulate FSHR levels, enhancing GCs proliferation, fostering follicle development, and ultimately improving ovarian function and reproductive outcomes. A new study (Lin et al., 2019) introduced the processing method of nine steamed and nine baked RRP, comparing it with three other RR preparations. Results indicated that the proliferation effect on rat ovarian GCs was associated with RR polysaccharide concentration, with the most significant effect at a mass concentration of 150 μg/mL. Among these, the total polysaccharide concentration was highest in RR processed by nine steamed and nine baked RRP, with the proliferation hierarchy as follows: nine steamed and nine sun-dried RRP polysaccharides > nine steamed and nine baked RRP polysaccharides > modern method RRP polysaccharides > RRR polysaccharides. Intriguingly, experimental studies (Liu, 2018) found that the aqueous extract of the modern steaming RRP method may hinder ovulation in normal female rats, leading to prolonged estrous cycles, increased atretic follicles, and decreased serum sex hormone (estradiol, progesterone) levels, while the aqueous extract of the nine steaming and nine sun-dried RRP may not affect ovulation in female rats with normal estrous cycle, but the specific compounds and dosage impacting ovulation require further investigation.

### 4.2 Antioxidant and anti-aging

Ovarian senescence, influenced by factors affecting the quantity and quality of oocytes, leads to a decline in ovarian function and gradual aging. In 1954, Harman first proposed the free radical theory of aging, suggesting that aging-related diseases are triggered by the interaction of reactive oxygen species (ROS) with cellular components, thereby inducing aging-related changes (Harman, 1956; Harman, 2006). Hence, reducing oxidative stress and the production of free radicals can decelerate aging and extend life. Oxidative stress is considered a principal pathogenesis of ovarian aging. Normally, a dynamic balance exists between ROS and antioxidants in the body. Disruption of this balance due to excessive ROS production or increased antioxidant utilization induces oxidative stress. Oxidative stress can lead to various pathological changes in cells, including mitochondrial dysfunction, DNA damage, telomere shortening, etc., all of which are key factors in aging-related diseases. Studies have shown that oxidative stress within the ovarian microenvironment can diminish oocyte quality, induce apoptosis of GCs, accelerate corpus luteum degeneration, and lead to ovarian aging and infertility (Freitas et al., 2017; Liang J. et al., 2023), affecting the success rate of ART (Oyawoye et al., 2003).

Excessive ROS can also initiate crosstalk among various signaling pathways and protein factors, contributing to the pathogenesis of ovarian aging and serving as therapeutic targets. Currently, the regulation of ovarian signaling pathways by ROS mainly focuses on important signaling pathways such as Recombinant Kelch Like ECH Associated Protein 1 (KEAP1)-Nuclear factor-E2-related factor 2 (Nrf2), NF-kB, FOXO, etc. Nrf2 is a critical transcription factor in oxidative stress, regulating the antioxidant response with KEAP1 to protect cellular functions (Baird and Yamamoto, 2020). Nrf2 promotes the expression of downstream antioxidant enzymes such as heme oxygenase 1 (HO-1), NADP(H) quinone dehydrogenase 1 (NQO1), and glutathione-S-transferases (GSTs). This mechanism effectively mitigates intracellular oxidative stress and minimizes cellular damage from ROS(Gao X. et al., 2023). Research has shown that (Ma et al., 2018) Nrf2 expression levels in ovarian GCs correlate with women’s age, highlighting Nrf2 as a key factor in oocyte aging and suggesting that a decline in Nrf2 expression may be closely linked to reduced reproductive capability in older women. Additionally, Nrf2 activity has been found to correlate positively with species longevity (Lewis et al., 2015) and improves endothelial cell senescence (Romero et al., 2019). Therefore, Nrf2 is one of the key points for treating ovarian aging. ROS induces inflammatory cells, such as macrophages, to produce TNF-α. When TNF-α binds to membrane receptors, it mediates the phosphorylation and degradation of IkB, activating the NF-kB pathway to control the transcription of anti-apoptotic and inflammation-related genes. The FOXO family, a crucial transcription regulatory factor within the PI3K-AKT signaling pathway, is involved in regulating cell proliferation, apoptosis, and differentiation. FOXO3 enhances NRF2 activity by promoting KEAP1 degradation and also increases BCL10 expression, regulating the activation of the IkB-NF-kB pathway (Shixuan, 2021).

In RR studies, processing has been found to increase the concentration of 5-HMF, a Maillard reaction product, enhancing free radical scavenging capabilities and playing an antioxidant role (Kwon et al., 2019). RR polysaccharide activates the Nrf2/Keap1 pathway and significantly increases the activity of antioxidant enzymes (Ren et al., 2023). However, Rehmannioside A notably improves oxidative stress after activating the PI3K/Nrf2/SLC7A11 signaling pathway (Fu et al., 2022). Additionally, the phenolic/phenylpropionic acid pathway, mediated by Cinnamate 4-hydroxylase (C4H), has been identified in RR phenol studies as involved in regulating oxidative stress tolerance (Yang Y. H. et al., 2021). In aging research, Rehmannia glutinosa 70 (RG70) has shown superior antioxidant activity *in vitro* (compared to RG50, RG90, and RGB), enhancing the antioxidant enzyme system of *Caenorhabditis elegans*, reducing ROS levels, and increasing the expression of lifespan-related genes daf-16 and skn-1 and their downstream genes sdo-3 and gcs-1, thereby achieving an anti-aging effect (Liang L. et al., 2023). Another study found that RG70 reduced the expression levels of TNF-α, IL-1β, and IL-6 by modulating the ROS/NF-kB signaling pathway, thereby alleviating oxidative stress and inflammatory response (Zhang H. et al., 2023). Another aspect of the anti-aging mechanism of RR is to enhance the function of hematopoietic stem cells, downregulate aging-related proteins p53 and p16, reduce ROS levels, and achieve an anti-aging effect (Bai et al., 2018). RR polysaccharide also activates the antioxidant enzyme system under oxidative stress, thereby stimulating the daf-16 gene on the insulin/IGF-1 signaling pathway (IIS) and prolonging the lifespan of *C. elegans* (Yuan et al., 2019). Moreover, some studies have found that microplastics activate the Wnt/β-Catenin signaling pathway and oxidative stress in rats, leading to apoptosis of ovarian GCs and causing fibrosis, ultimately reducing ovarian reserve function (An et al., 2021). In a study of RR extract catalpol, it was found to regulate pulmonary fibrosis through the Wnt/β-catenin pathway and reduce oxidative stress in lung tissue (Yang F. et al., 2021). Another experiment with the RR-Cornus officinalis pair revealed that this combination could reduce pro-inflammatory cytokines IL-1β, IL-6, and TNF-α, and downregulate the expression of TGF-β1, JNK, p38, and ERK to improve renal interstitial fibrosis (Zhu et al., 2024). Future studies could also explore the pharmacological mechanism of RR in ameliorating ovarian aging from the perspective of ovarian fibrosis.

### 4.3 Regulates the immune inflammatory microenvironment

The ovary is frequently targeted by autoimmune attacks in both organ-specific and systemic autoimmune diseases. The precise mechanism of autoimmunity in these conditions remains unclear, potentially triggered by genetic or environmental factors (Domniz and Meirow, 2019). Studies indicate that between 10% and 55% of patients with POI also suffer from autoimmune diseases (Szeliga et al., 2021). In cases of POI with adrenal autoimmune disease, ovarian histology biopsies reveal persistent autoimmune oophoritis, with inflammatory cells particularly surrounding the pre-ovulatory follicle and corpus luteum which produce steroid hormones (Kirshenbaum and Orvieto, 2019). Research has demonstrated a disorder in T lymphocyte subsets in POI patients. Compared to healthy women, POI patients show a decreased percentage of CD4^+^ T lymphocytes and an increased percentage of CD8^+^ T lymphocytes in peripheral blood, with TSH levels negatively correlated with CD4^+^ percentages and positively with CD8^+^ percentages (Hsieh and Ho, 2021). Additionally, the expression of the fork-head transcription factor 3 (Foxp3) in peripheral blood is significantly reduced in POI patients, alongside increased levels of pro-inflammatory factors such as interferon γ (IFN-γ) and TNF-α, and decreased levels of anti-inflammatory factors like interleukin-10 (IL-10) and transforming growth factor beta (TGF-β) (Wang et al., 2022; Mao and Ji, 2023). Th1/Th2 cells, subtypes of CD4^+^ T cells, play roles in mediating cellular immune and inflammatory responses, secreting pro-inflammatory cytokines such as IFN-γ, TNF-α, and IL-6, while Th2 cells primarily secrete anti-inflammatory cytokines like TGF-β and IL-10. These studies suggest a disturbance in the local immune inflammatory microenvironment in POI patients, with an imbalance of Th1/Th2 cells in the ovaries disrupting homeostasis, affecting follicle formation and ovulation (Boots and Jungheim, 2015), potentially leading to apoptosis and follicular atresia (Huang et al., 2019). Moreover, the local inflammatory state can induce ovarian fibrosis, making modulation of the immune inflammatory microenvironment another therapeutic approach for treating ovarian aging.

Extensive research confirms that immunity regulation and anti-inflammation are among the pharmacological effects of RR. RR polysaccharides, particularly after repeated steam drying, effectively enhance anti-inflammatory activity (Lu et al., 2022). These polysaccharides activate dendritic cells (DCs) (Huang et al., 2013) both *in vitro* and *in vivo*, increase cell proliferation and cytokine secretion, and sustain cellular and humoral immune responses (Huang et al., 2016a). They promote IFN-γ production in CD4^+^ and CD8^+^ T cells (Xu et al., 2017) and increase Th1, Th2, and Th17 cytokines in mice, thus improving immunity (Huang et al., 2021). RR polysaccharides also activate the non-specific immune response of macrophages by increasing nitric oxide levels, enhancing macrophage phagocytic activity (Huang et al., 2016b), and thereby bolstering immune response and anti-infection capabilities (Feng et al., 2020). Additionally, 2,5-dihydroxyacetylbenzone (DHAP), extracted from RRP, has shown potential in treating lipopolysaccharide (LPS)-stimulated macrophage inflammatory responses in RAW264.7 mice. DHAP effectively inhibited the phosphorylation of extracellular signal-related kinase (ERK) 1/2 and nuclear translocation of NF-κB p65, reducing inflammatory mediator production in activated macrophages and achieving anti-inflammatory activity (Han et al., 2012). Recent studies also link gut microbiota with POI (Wu et al., 2021), with Mendelian studies suggesting that dysbiosis of gut microbiota can lead to POI (Wang et al., 2023). Notably, research on RR oligosaccharides and polysaccharides has shown they can enhance the abundance of intestinal microbiota, reduce intestinal inflammation, and repair intestinal barrier damage by maintaining intestinal microbiota homeostasis (Li X. et al., 2023; Lv et al., 2023; Ren et al., 2023). Furthermore, RR oligosaccharides enhance the vitality of stem cells and improve the immunomodulatory efficacy of stem cells, achieving better therapeutic outcomes (Zhang et al., 2012). Interestingly, in a study of overweight women, RR was found to reduce BMI by improving gut microbiota, enriching research on RR in metabolic diseases and underscoring its potential as a prebiotic (Han et al., 2015).

### 4.4 Impact on long-term complications: cardiovascular and cerebrovascular diseases, osteoporosis, and cognitive dysfunction

Estrogen can inhibit macrophage oxidation of low-density lipoprotein, induce direct antioxidant effects to reduce macrophage activation through oxidized low-density lipoprotein protein and prevent the progression of atherosclerosis (Clarkson, 2018). Estrogen can also directly act on cardiomyocytes, including activating the AKT pathway and inhibiting cardiomyocyte apoptosis and necrosis. Patients with diseases of ovarian hypofunction diseases are at increased risk of cardiovascular and cerebrovascular diseases due to decreased estrogen levels. The risk of cardiovascular and cerebrovascular diseases increases with age. A Post-menopause, women experience prolonged low estrogen levels, reducing the protective effects on blood vessels and heightening the risk of cardiovascular and cerebrovascular diseases compared to men of the same age (Bushnell et al., 2014). The ovarian hormone 17β-estradiol plays a critical role in sex differences in cardiovascular diseases due to its impact on myocardial remodeling, function, and atherosclerotic lesion management (Bourassa et al., 1996; Stewart et al., 2006). A meta-analysis (Daan et al., 2016) revealed that women with POI face higher cardiovascular risks than middle-aged premenopausal women, including increased abdominal fat, elevated chronic inflammatory markers, and tendencies towards higher blood pressure and impaired kidney function. RR extract, particularly catalpol, has been found to protect rat cardiomyocytes by regulating autophagy, inhibiting apoptosis, enhancing oxidative stress response, and modulating estrogen receptors, thereby improving myocardial ischemia (Lin et al., 2017). Catalpol also mitigates oxidative stress, inflammatory responses, and apoptosis in the heart by inhibiting NF-κB activation, reducing cardiovascular event occurrences (Nemmar et al., 2022). Hyperhomocysteinemia (HHCY), a recognized independent risk factor for atherosclerosis, is ameliorated by catalpol in human aortic endothelial cells through the inhibition of Nox4/NF-κB and endoplasmic reticulum stress (Hu et al., 2019). Additionally, the active compound puerarin from Catalpol and Pueraria lobata significantly improves cerebrovascular endothelial cell apoptosis, increases local cerebral blood flow, and reduces infarct size at a dosage of 65.4 mg/kg (Liu et al., 2017).

Premature ovarian aging leads to decreased bone mineral density and increased osteoporosis risk, partly due to the adverse effects of prolonged high FSH levels and low estrogen (Mills et al., 2021). A longitudinal study (Shea et al., 2021) indicated a higher osteoporosis incidence in women with POI compared to those with early or normal menopausal onset (21.9% vs. 16.7%). Extensive research has shown that RR extract effectively prevents and treats osteoporosis, potentially developing as a new therapeutic drug. This effect is attributed to RR extract’s ability to stimulate osteoblast proliferation, inhibit osteoclast activity and production, and prevent bone loss in osteoporotic mouse models with ovarian resection (Oh et al., 2003; Lim and Kim, 2013). Inosine, a primary active molecule in RR, significantly inhibits bone marrow macrophage-derived osteoclast differentiation and formation by blocking NF-κB activation, thereby reducing bone loss (Lee et al., 2013). RR compound ajugol alleviates osteoarthritis by promoting autophagy and attenuating endoplasmic reticulum stress-induced cell death and extracellular matrix degradation at 50 μM(Wu et al., 2023). Metabolomics studies (Xia et al., 2019) reveal that RR prevents dexamethasone-induced bone loss by interfering with steroid hormone biosynthesis, upregulating cytochrome P450 17A1 (CYP17A1) and aromatase (CYP19A1), and downregulating 11β-hydroxysteroid dehydrogenase (HSD11B1).

Cognitive dysfunction and memory loss are also significant long-term risks after premature ovarian failure, closely linked to estrogen deficiency (Gibbs, 2010; Djiogue et al., 2018). Notably, a meta-analysis (Zhao et al., 2023) indicated that osteoporotic patients have a higher risk of cognitive impairment compared to non-osteoporotic patients (OR = 2.01%, 95% CI: 1.63–2.48, *p* < 0.01). Catalpol isolated from RR plays a neuroprotective role in brain tissues and improves cognitive dysfunction by enhancing endogenous antioxidant enzyme activity and inhibiting free radical production (Zhang et al., 2007). Another study (Zhang et al., 2013) found that Catalpol also offers neuroprotective effects in d-galactose-induced aging mice by regulating the cholinergic system and reducing inflammatory cytokine expression (TNF-α, IL-1β). Thus, the active compounds of RR effectively reduce long-term health risks in patients with premature ovarian aging and offer benefits for both short-term and long-term health.

## 5 The application of TCM compound with RR in ovarian hypofunction diseases

RR is commonly used in clinical practice in combination with other Chinese botanical drugs in recipes such as Siwu Decoction, Liuwei Dihuang Pill, Guishen Pill, Yijing Decoction, and Chinese patent medicines like KunTai Capsule. As the primary botanical drug in many TCM compounds, RR can enhance the efficacy of these formulas. The composition of TCM compounds containing RRP was shown in Table 2, and its mechanism were shown in Table 3 and Table 4.

**TABLE 2:** Basic research on traditional Chinese medicine compounds of Rehmanniae Radix.

Recipe	Disease	Animals/Cells	Dosage	Treatment targets	Mechanisms	Reference
Siwu Decoction	POF	Mice	3.24 g/(kg·d) 12.96 g/(kg·d)	Promoting the production of VEGF and bFGF, and regulating the expression of PDGFB, Ang1, and Ang2	Enhancing ovarian angiogenesis in POF mice, maintaining the stability and maturation of newly formed blood vessels and supporting follicle development	Zhou (2022)
	NR	Rats	1.04 g/(kg·d) 2.08 g/(kg·d)	Upregulating the expression of ERα and ERβ, and reducing the ratio of ERα/ERβ	Exerting estrogen-like effects, increasing uterine tissue weight, and promoting uterine development	Lu et al. (2019)
		Human breast cancer cells MCF7	NR			
	DOR	Rats	5.4 g/(kg·d) 10.8 g/(kg·d)	increasing the abundance of Verrucomicrobia, Epsilonbacteraeota, and Christensenellaceae, and decreasing the abundance of Firmicutes and Bacteroidetes	Influencing intestinal flora, increase in beneficial bacteria, regulating energy metabolism, biological repair, and synthesis, improving ovarian function, and decreasing FSH and LH, increasing E_2_	Zhu et al. (2022b)
Liuwei Dihuang Pill	DOR	Rats	1.08 g/(kg·d)	Upregulating the expression of YY1 and downregulating the expression of CYP4F3	Protecting the integrity of the mitochondrial morphology of ovarian granulosa cells, inhibiting oxidative stress, promoting follicle development, Increasing litter size and survival rate of offspring	Gao et al. (2023b)
	ODI	Granulosa cells	NR	Increase in the number of AGNORs	Proliferation of ovarian granulosa cells and promoting follicle development	Yue (2009)
	OP	Rats	0.1575 g/(kg·d)	Downregulating the expression of Caspase-1, IL-1β and TNF-α in the serum and reducing the mRNA expression of NLRP3 and GSDMD in bone tissue	Inhibiting inflammatory factors, alleviating inflammatory response, regulating cell pyroptosis, and resisting osteoporosis	Chen et al. (2023b)
Guishen Pill	DOR	Mice	21.6 g/(kg·d)	Upregulating the expression of ER, PR, MVH, Oct-4 and Egr1	Influencing the secretion of estrogen and AMH in females, improving ovarian function	Cui et al. (2015)
	DOR	Mice	1.4 g/(kg·d) 2.8 g/(kg·d) 5.6 g/(kg·d)	Upregulating the expression of LC3 and Beclin 1, Downregulating the expression of p62 in Ovarian tissue	Reversing the Cyclophosphamide -induced ovarian tissue excessive autophagy state, promoting the dynamic balance of autophagy, improving hormone levels, and restoring ovarian function	Zhu and Du (2023)
	POF	Mice	12 g/(kg·d) 24 g/(kg·d) 48 g/(kg·d)	Increasing IFN-γ and IL-2 levels significantly, elevating the number of CD3^+^ T cells, CD4^+^ T cells and NK cells, and raising CD4^+^ T/CD8^+^ T ratio	Increasing the spleen index of mice, enhancing the proliferation ability of T cells, regulating the balance of T cell subsets, and improving immune function disorders caused by premature ovarian failure	Li et al. (2018)
	POF	Rats	9.45 g/(kg·d)	Downregulating the expression of RANKL, upregulating the expression of OPG in bone tissue, promoting CD4^+^CD25^+^Foxp3^+^Treg cells, and increasing the level of IL-10 and TGF-β in the serum	Inhibiting the differentiation and activity of osteoclasts, improving the bone loss and microstructural damage of femurs in POF rats, and having a therapeutic effect on osteoporosis	Ba et al. (2023)
Yijing Decoction	DOR	Rats	5.9 g/(kg·d) 11.8 g/(kg·d) 23.6 g/(kg·d)	Downregulating the expression of HIF-1α, Bnip3 and Beclin1	Improving the hypoxic state of DOR model rats, reversing the process of cellular autophagy, regulating hormone secretion, and protecting ovarian tissues	Wan and Chen (2022)
	DOR	Rats	11.79 g/(kg·d)	Downregulating the level of TNF-α and IFN-γ, downregulating the expression of SDF-1 and CXCR4, and reducing the proportion of Th17 cells and increasing the proportion of Treg cells	Regulating the Th17 cells/Treg cells balance, alleviating ovarian immune inflammation damage, decreasing the levels of FSH, LH, and IL-17 in serum, increasing the levels of E_2_, AMH, and IL-10, and enhancing ovarian index and uterine index	Xie (2021)
Kuntai Capsule	POI	Mice	0.4 g/(kg·d) 0.8 g/(kg·d) 1.6 g/(kg·d)	Upregulating the expression of SIRT5 and FOXO3a proteins	Reducing ROS, enhancing antioxidant enzyme activity, regulating mitochondrial oxidative stress, and participating in cell proliferation and the cell cycle	Gong et al. (2024)
	POI	Rats	0.6 g/(kg·d) 1.8 g/(kg·d)	Upregulating the expression of LC3A-II, GDF9 and Beclin-1	Regulating autophagy, inhibiting or partnering with apoptosis, eliminating damaged organelles to adapt to constantly changing internal and external environments, and promoting cell survival by delaying aging and cell death	Luo et al. (2023)
	DOR	Rats	0.21 g/(kg·d) 0.63 g/(kg·d) 1.89 g/(kg·d)	Upregulating the expression of Bcl-2 protein, downregulating the expression of Bax protein, and raising the ratio of Bcl-2 and Bax	Inhibiting the apoptosis of ovarian granulosa cells, delaying aging, downregulating FSH and LH levels, and upregulating E_2_ levels	Geng and Tan (2017)

P.S: Not reported (NR); Premature ovarian failure (POF); Premature ovarian insufficiency (POI); Diminished ovarian reserve (DOR); Osteoporosis (OP); Ovulatory disorder infertility (ODI); Reactive oxygen species (ROS); Follicle stimulating hormone (FSH); Luteinizing hormone (LH); Estradiol (E_2_); Anti-Müllerian hormone (AMH).

**TABLE 3:** Clinical research on traditional Chinese medicine compounds of Rehmanniae Radix.

Recipe	Author & years	Disease	Samples		Intervention		Duration		Outcomes	Reference
			T	C	T	C	T	C		
Liuwei Dihuang Pill	Gao L 2021	DOR	33	33	Liuwei Dihuang Pill + HRT	HRT	3 months	3 months	FSH, LH, E_2_, AMH, AFC, OV	Gao (2021)
	Qin LH 2017	POF	39	39	Liuwei Dihuang Pill + HRT	HRT	3 months	3 months	FSH, LH, E_2_	Qin (2017)
	Li Q 2016	POF	61	48	Liuwei Dihuang Pill + HRT	HRT	6 months	6 months	FSH, LH, E_2_	Li et al. (2016)
	Du JM 2016	POF	40	40	Liuwei Dihuang Pill + HRT	HRT	3 months	3 months	FSH, LH, E_2_	Du (2016)
	Du D 2013	POF	32	30	Liuwei Dihuang Pill + HRT	HRT	3 months	3 months	FSH, LH, E_2_	Du et al. (2013)
Guishen Pill	Xu JX 2020	DOR	60	60	Guishen Pill + HRT	HRT	3 months	3 months	FSH, LH, E_2_, AFC, OV, Pregnancy rate, PSV, Adverse reaction	Xu et al. (2020)
	Hu XH 2023	POF	43	43	Guishen Pill + HRT	HRT	21 days	21 days	FSH, LH, E_2_	Hu et al. (2023)
	Du QM 2018	POF	30	30	Guishen Pill + HRT	HRT	6 months	6 months	FSH, E_2_, PSV	Du and Fan (2018)
Yijing Decoction	Li LM 2017	DOR	33	34	Yijing Decoction	HRT	3 months	3 months	FSH, LH, E_2_, AMH, AFC, Adverse reaction	Li et al. (2017)
	Zhang JM 2023	POF	48	48	Yijing Decoction + HRT	HRT	6 weeks	6 weeks	FSH, LH, E_2_, P, T, PRL	Zhang and Lin (2023)
	Hong YL 2019	POR	33	33	Yijing Decoction + PPOS	PPOS	3 months	-	FSH, E_2_, AMH, AFC, Mature follicle rate, Embryo quality rate	Hong et al. (2019)
Kuntai Capsule	Gao P 2019	DOR	40	40	Kuntai Capsule	HRT	9 weeks	3 months	FSH, LH, E_2_, PSV	Gao (2019)
	Lin XM 2021	DOR	65	65	Kuntai Capsule	HRT	3 months	3 months	FSH, E_2_, AMH, AFC, PSV	Lin et al. (2021)
	Cui N 2018	DOR	33	35	Kuntai Capsule	Dehydroepiandrosterone	3 months	3 months	FSH, LH, E_2_, AMH, AFC, Pregnancy rate, Adverse reaction P, T, PRL	Cui et al. (2018)
	Zhou XH 2023	POF	43	43	Kuntai Capsule + HRT	HRT	3 months	3 months	FSH, LH, E_2_, AMH, AFC, OV, Endometrial thickness, Adverse reaction	Zhou et al. (2023)
	Jiang LL 2023	POF	43	43	Kuntai Capsule + HRT	HRT	12 weeks	12 weeks	FSH, LH, E_2_, AMH, AFC, OV, Treg/%, Th17/%, TGF-β1, IL-21	Jiang et al. (2023b)

P.S: Not reported (NR).

Disease: Premature ovarian failure (POF); Premature ovarian insufficiency (POI); Diminished ovarian reserve (DOR); Poor ovarian response (POR).

Intervention: Hormone replacement therapy (HRT); MPA + Human menopausal gonadotropin (HMG)+ Human chorionic gonadotrophin (HCG)+ GnRH-a (PPOS).

Outcomes: Follicle stimulating hormone (FSH); Luteinizing hormone (LH); Estradiol (E_2_); Anti-Müllerian hormone (AMH); Antral follicle count (AFC); Ovarian volume (OV); Peak systolic velocity (PSV); Progesterone (P); Testosterone (T); Prolactin (PRL).

**TABLE 4:** The composition of traditional Chinese medicine compounds containing Rehmanniae Radix Praeparata.

Recipe	Composition			Medicinal part
	Scientific name	Species name	Name in pharmacopeia	
Siwu decoction	Rehmannia glutinosa (Gaertn.) DC	Orobanchaceae	Rehmanniae Radix Praeparata	Dried tuber roots
	Angelica sinensis (Oliv.) Diels	Apiaceae	Angelicae Sinensis Radix	Dried roots
	Paeonia lactiflora Pall	Paeoniaceae	Paeoniae Radix Alba	Dried roots
	Conioselinum anthriscoides ‘Chuanxiong'	Apiaceae	Chuanxiong Rhizoma	Dried rhizomes
Liuwei Dihuang Pill	Rehmannia glutinosa (Gaertn.) DC	Orobanchaceae	Rehmanniae Radix Praeparata	Dried tuber roots
	Dioscorea oppositifolia L	Dioscoreaceae	Dioscoreae Rhizoma	Dried rhizomes
	Cornus officinalis Siebold & Zucc	Cornaceae	Corni Fructus	Dried ripe pulp
	Poria cocos (Schw.)Wolf		Poria	Dried sclerotium
	Alisma plantago-aquatica subsp. orientale (Sam.) Sam	Alismataceae	Alismatis Rhizoma	Dried stem tuber
	Paeonia × suffruticosa Andrews	Paeoniaceae	Moutan Cortex	Dried root bark
Guishen Pill	Rehmannia glutinosa (Gaertn.) DC.	Orobanchaceae	Rehmanniae Radix Praeparata	Dried tuber roots
	Dioscorea oppositifolia L	Dioscoreaceae	Dioscoreae Rhizoma	Dried rhizomes
	Cornus officinalis Siebold & Zucc	Cornaceae	Corni Fructus	Dried mature pulp
	Poria cocos (Schw.)Wolf	Polyporaceae	Poria	Dried sclerotium
	Angelica sinensis (Oliv.) Diels	Apiaceae	Angelicae Sinensis Radix	Dried roots
	Lycium barbarum L	Solanaceae	Lycii Fructus	Dried ripe fruit
	Eucommia ulmoides Oliv	Eucommiaceae	Eucommiae Cortex	Dried Bark
	Cuscuta chinensis Lam	Convolvulaceae	Cuscutae Semen	Dried ripe seeds
Yijing decoction	Rehmannia glutinosa (Gaertn.) DC.	Orobanchaceae	Rehmanniae Radix Praeparata	Dried tuber roots
	Atractylodes macrocephala Koidz	Asteraceae	Atractylodis Macrocephalae Rhizoma	Dried rhizomes
	Dioscorea oppositifolia L	Dioscoreaceae	Dioscoreae Rhizoma	Dried rhizomes
	Angelica sinensis (Oliv.) Diels	Apiaceae	Angelicae Sinensis Radix	Dried roots
	Paeonia lactiflora Pall	Paeoniaceae	Paeoniae Radix Alba	Dried roots
	Ziziphus jujuba Mill	Rhamnaceae	Jujubae Fructus	Dried ripe fruit
	Paeonia × suffruticosa Andrews	Paeoniaceae	Moutan Cortex	Dried root bark
	Adenophora triphylla (Thunb.) A.DC	Campanulaceae	Adenophorae Radix	Dried roots
	Bupleurum chinense DC	Apiaceae	Bupleuri Radix	Dried roots
	Eucommia ulmoides Oliv	Eucommiaceae	Eucommiae Cortex	Dried Bark
	Panax ginseng C.A.Mey	Araliaceae	Ginseng Radix Et Rhizoma	Dried roots and rhizomes
Kuntai Capsule	Rehmannia glutinosa (Gaertn.) DC	Orobanchaceae	Rehmanniae Radix Praeparata	Dried tuber roots
	Coptis chinensis Franch	Ranunculaceae	Coptidis Rhizoma	Dried rhizomes
	Paeonia lactiflora Pall	Paeoniaceae	Paeoniae Radix Alba	Dried roots
	Scutellaria baicalensis Georgi	Lamiaceae	Scutellariae Radix	Dried roots
	Equus asinusl	Equine	Asini Corii Colla	Dried donkey skin or fresh skin by boiling, concentrated into a solid glue
	Poria cocos (Schw.)Wolf	polyporaceae	Poria	Dried sclerotium

PS: The “Scientific name” and “Species name” in the table were retrieved from the “Medicinal Plant Names Services” website (https://mpns.science.kew.org/mpns-portal); “Name in pharmacopeia” was retrieved from *National Pharmacopoeia Committee. Pharmacopoeia of the People’s Republic of China: Volume I*. among them, the appropriate “Scientific names” and “Species name” of Poria and Asini Corii Colla could not be retrieved from the website and were searched from the pharmacopoeia instead.

### 5.1 Siwu decoction

Siwu Decoction, in which RRP is as its sovereign botanical drug, Angelicae Sinensis Radix as the minister botanical drug, and Paeoniae Radix Alba and Chuanxiong Rhizoma as assistant botanical drugs, serves to nourish and replenish blood. As a tonic formula, it was first documented in the Song Dynasty’s “*Taiping Huimin Heji Ju Fang*” for treating gynecological conditions. Today, it is widely employed to address irregular menstruation and various complications during pregnancy and childbirth, including those associated with ovarian hypofunction. Each menstrual cycle involves cyclic ovarian remodeling, necessitating extensive vascular remodeling to support new follicle growth. A dense, highly permeable vascular network supplies the ovary with essential hormones and nutrients for robust metabolism and delivers matrix factors to the cumulus-oocyte complex before ovulation (Brown and Russell, 2014). Consequently, ovarian blood vessels are essential for follicles, ovarian structure, and function. Research on Siwu Decoction has demonstrated its ability to enhance ovarian angiogenesis in POF model mice by activating the STAT3/HIF-1a/VEGF signaling pathway, thus increasing the production of related pro-angiogenic factors (VEGF, bFGF). It also stabilizes new blood vessel maturation through the regulation of PDGFB, Ang1, and Ang2 expression, ensuring sufficient vascular support for follicle development and consistent proliferation, and enhancing ovarian function (Zhou, 2022). Estrogen receptors (ER), including ERα, ERβ, and G protein-coupled estrogen receptor 1 (GPER), are crucial for follicle and oocyte growth, development, and ovulation (Tang et al., 2019). Phytoestrogens, natural compounds with significant ER regulatory activity, offer alternatives for those unwilling or unable to undergo HRT (Yang et al., 2019). *In vitro* cell studies have shown that serum containing Siwu Decoction exhibits estrogen-like effects, potentially through regulation of ERα and ERβ expression (Lu et al., 2019). Earlier discussions highlighted the findings from Mendelian Randomization (MR) studies on the link between POI and intestinal microbiota. Studies have shown that DOR rats induced by Tripterygium glycosides exhibit reduced intestinal microbiota diversity. Yet, following treatment with Siwu Decoction, there is a significant recovery in the diversity of intestinal microbiota in model rats, with a concurrent increase in beneficial bacteria (Zhu M. et al., 2022).

### 5.2 Liuwei Dihuang Pill

Liuwei Dihuang Pill comprises six botanical drugs: RRP, Dioscoreae Rhizoma, Corni Fructus, Poria, Alismatis Rhizoma, and Moutan Cortex. This formula is a fundamental TCM preparation for kidney nourishment and yin reinforcement, commonly employed in treating ovarian hypofunction diseases. Clinical trials (Du et al., 2013; Du, 2016; Li et al., 2016; Qin, 2017; Gao, 2021) have demonstrated that combining Liuwei Dihuang Pill with HRT enhances clinical symptoms and sex hormone levels (FSH, LH, E_2_) more effectively than HRT alone, without increasing adverse reactions and potentially boosting the therapeutic outcomes. Fundamental research has shown that Liuwei Dihuang Pill promotes the proliferation of human ovarian granulosa cells and follicle maturation, thus facilitating ovulation, making it suitable for treating ovulatory disorders diseases (Yue, 2009). Another study revealed that Liuwei Dihuang Pill protects the mitochondrial integrity of ovarian granulosa cells and ameliorates mitochondrial dysfunction in cyclophosphamide-induced DOR mouse models. This protective effect is achieved by reducing ROS accumulation, mitigating oxidative stress, and thus preventing granulosa cell apoptosis (Gao T. et al., 2023). Subsequent research on these model mice indicated that intervention with Liuwei Dihuang Pill results in a higher number of offspring and pup survival rates compared to the control group. Furthermore, studies on long-term health risks suggest that Liuwei Dihuang Pill may reduce inflammatory responses and regulate bone metabolism via modulation of the NLRP3/Caspase-1/GSDMD pyroptosis signaling pathway, offering potential anti-osteoporosis benefits (Chen ZB. et al., 2023).

### 5.3 Guishen Pill

The Guishen Pill contains RRP, Dioscoreae Rhizoma, Corni Fructus, Poria, Angelicae Sinensis Radix, Lycii Fructus, Eucommiae Cortex, and Cuscutae Semen. This formula nourishes kidney essence, tonifies blood, and regulates menstruation. Guishen Pill, a TCM formula for tonifying the kidney and benefiting essence, is commonly used to treat ovarian hypofunction. Clinical studies (Du and Fan, 2018; Xu et al., 2020; Hu et al., 2023) indicate that Guishen Pill, when combined with HRT, surpasses HRT alone in enhancing endometrial thickness, ovarian volume, and sex hormone levels in patients with ovarian hypofunction diseases, without exacerbating adverse effects. Oxidative stress damage is a primary aging factor. An experiment (Cui et al., 2015) developed an ovarian aging model in mice through continuous superovulation and ozone inhalation, intensifying oxidative stress and diminishing follicle quality. Post-intervention with Guishen Pill led to increased expression of Oct-4 and MVH mRNA in model mice (*p* < 0.05). Oct-4 is identified with embryonic-like stem cells and ovarian germ cells (Johnson et al., 2005; Virant-Klun, 2015). Research reveals that mice deficient in the MVH gene show arrested differentiation and apoptosis in reproductive cells, marked by reduced Oct-4 expression, resulting in compromised reproductive capacity (Tanaka et al., 2000). Therefore, it can be speculated that Guishen Pill may improve stem cell function by inhibiting oxidative stress, thereby promoting the differentiation of oocyte cells and restoring ovarian function in mice. Additionally, animal experiments have demonstrated that Guishen Pill can reduce the protein and mRNA levels of LC3 and Beclin 1 in the ovarian tissues of DOR mice, increase the levels of p62 protein and mRNA, and improve ovarian function by inhibiting ovarian autophagy (Zhu and Du, 2023). Meanwhile, in studying the immune mechanisms in ovarian premature aging model mice, it was found that Guishen Pill could significantly increase the spleen index, enhance the proliferation ability of T cells, significantly increase the ratio of CD3^+^T, CD4^+^T, CD4^+^T/CD8^+^T, NK cells, IFN-γ and IL-2 in mice (*p* < 0.05), and significantly decrease the percentage of CD8^+^ T cells (*p* < 0.05) (Li et al., 2018). Moreover, Guishen Pill can also improve bone mass and reduce microstructural damage in the femurs of rats with POF, thereby achieving its potential in treating osteoporosis (Ba et al., 2023).

### 5.4 Yijing decoction

Yijing Decoction, derived from *Fu Qingzhu’s Obstetrics and Gynecology*, contains eleven medicinal ingredients: RRP, Atractylodis Macrocephalae Rhizoma, Dioscoreae Rhizoma, Angelicae Sinensis Radix, Paeoniae Radix Alba, Jujubae Fructus, Moutan Cortex, Adenophorae Radix, Bupleuri Radix, Eucommiae Cortex and Ginseng Radix Et Rhizoma. The book employs this formula to treat patients who appear amenorrhea before age 49″, which aligns with the characteristics of ovarian hypofunction diseases. Subsequent clinical studies (Li et al., 2017; Zhang and Lin, 2023) demonstrate that Yijing Decoction improves clinical symptoms and sex hormone levels of patients with ovarian hypofunction diseases, thus enhancing pregnancy outcomes. For older patients with diminished ovarian reserve undergoing IVF-ET, Yijing Decoction, used alongside controlled ovarian stimulation protocols, significantly increases the number of retrieved ova, maturity of ova, and quality of embryos, improving ovarian responsiveness and oocytes and embryo quality (Hong et al., 2019). Experimental research (Wan and Chen, 2022) shows that Yijing Decoction counteracts hypoxia-induced activation of the Bnip3/Beclin1 pathway, thereby reducing autophagy-induced damage to ovarian tissue. The SDF-1 (stromal cell-derived factor-1)/CXCR4 axis is pivotal in embryonic development and enhances vascular regeneration and the resolution of ischemic conditions in injured tissues. SDF-1 also serves as a crucial cytokine in the regulation of local inflammation and tissue repair (Cencioni et al., 2012). Research on Yijing Decoction has demonstrated that this formula can modulate the Th17/Treg balance through the SDF-1/CXCR4 pathway, decreasing IL-17 and increasing IL-10 levels. This mechanism alters the immune-inflammatory microenvironment, thereby affecting ovarian function in cyclophosphamide-induced DOR model rats (Xie, 2021).

### 5.5 Kuntai capsule

Kuntai capsule, derived from the HuangLian-EJiao decoction in the *Treatise on Febrile Diseases*, consist of RRP, Coptidis Rhizoma, Paeoniae Radix Alba, Scutellariae Radix, Asini Corii Colla, and Poria. The efficacy and safety has been confirmed through numerous clinical trials (Cui et al., 2018; Gao, 2019; Lin et al., 2021; Zhou et al., 2023) and systematic reviews (Shen et al., 2020; A RN et al., 2019; Zhang et al., 2024; Liu et al., 2021). A study (Gong et al., 2024) identified that a primary target of Kuntai capsule in treating POF model mice is the regulation of oxidative stress damage, primarily through the FOXO3 and SIRT5 genes. The AGE-RAGE signaling pathway plays a crucial role in managing oxidative stress and inflammation (Shi et al., 2023), and Kuntai capsule may enhance ovarian function by promoting follicular development and increasing the granulosa cell layer thickness in dominant follicles via this pathway (Huang et al., 2024). Further research (Luo et al., 2023) suggests that Kuntai capsule also boosts the expression of GDF9, a key TGF-β family member vital for granulosa cell proliferation and follicular growth. They also found that Kuntai capsule can upregulate the expression of Beclin 1, mediate cell autophagy, delay delayed cell senescence and cell death to promote cell survival. Regarding apoptosis, Kuntai capsule has been found to maintain the balance between pro-apoptotic and inhibitory proteins by adjusting the Bcl-2/Bax ratio, specifically by upregulating Bcl-2 and downregulating Bax expression, thus regulating apoptosis of ovarian granulosa cells to improve ovarian reserve function and delay aging (Geng and Tan, 2017). Moreover, the imbalance of the Th17/Treg cell ratio, a significant factor in immune disorders, is corrected in patients with ovarian hypofunction. Clinical studies have shown that Kuntai capsule increases TGF-β1 levels in the peripheral blood of POF patients, reduces IL-21 levels, and corrects the Th17/Treg cell ratio imbalance, thereby regulating the autoimmune system (Jiang LL. et al., 2023).

## 6 Limitations and future research prospects

Processing plays a critical role in influencing the pharmacological characteristics and effectiveness of botanical drugs. Various processing techniques can have diverse impacts on the bioactive compounds of herbal medicines, leading to modifications in their physiological effects. Consequently, the selection of an appropriate processing method to effectively manipulate the pharmacological properties of botanical drugs is essential for clinical applications. Nevertheless, regional disparities exist in the preparation methods of RRP in different regions. For example, regarding the processing method of prepared RRP, Zhu XD et al. (Zhu et al., 2023) used the “Jiangxi Provincial Standard for the Processing of Chinese Medicinal Slices (2008 Edition),” Chen QY et al. (Chen et al., 2021) used the “Jianchang Bang Processing Technique,” and Tian JY et al. (Tian et al., 2022) used the Beijing 2008 edition of the Drug Processing Specification. Some experiments even opted for purchasing RRP (Zhang et al., 2016). Furthermore, inconsistencies exist in the inclusion of excipients during RR steaming processes. For instance, Chen SQ et al. (Chen SQ. et al., 2023) utilized plain water for steaming RR, whereas Ma YJ et al. (Ma et al., 2023) employed yellow rice wine in the steaming process. These variations present difficulties in conducting quantitative analysis. In terms of compound analysis, the 2020 edition of the Pharmacopoeia of the People’s Republic of China only provides concentration specifications for catalpol and rehmannioside D in RRP and RR, without establishing concentration limits for other potentially bioactive compounds. Moreover, investigations into the use of RRP for conditions associated with ovarian hypofunction predominantly rely on findings from *in vitro* and *in vivo* studies, with limited incorporation of clinical research data. Clinical trials utilizing traditional Chinese medicine compounds containing RRP as the key component often exhibit small sample sizes, potentially compromising the applicability of their findings. Furthermore, the absence of blinding in many clinical studies, as well as their restricted focus on Asian populations, may introduce bias into the research outcomes.

Hence, it is imperative to enhance the following facets in forthcoming research: 1) standardizing and unifying the processing methods of RR and the addition of excipients, investigating the changes in RR compounds after adding excipients, and optimizing the storage conditions of botanical drugs according to the characteristics of processed products to guarantee their stability. 2) Conducting clinical research on the efficacious compounds of RRP for ovarian hypofunction involves integrating both basic and clinical research methodologies to substantiate their efficacy and safety, elucidating their scientific implications and mechanisms, and potentially developing RRP as pharmaceutical agents for the treatment of ovarian hypofunction. 3) Standardizing the clinical research design of traditional Chinese medicine compounds containing RRP aims to furnish high-quality evidence-based data for clinical application.
